# Role of Alternative and Augmentative Communication in Three Cases of Severe Acquired Brain Injury: A Neurorehabilitative Approach

**DOI:** 10.3390/brainsci14070709

**Published:** 2024-07-15

**Authors:** Caterina Formica, Maria Cristina De Cola, Francesco Corallo, Viviana Lo Buono

**Affiliations:** IRCCS Centro Neurolesi “Bonino-Pulejo”, 98124 Messina, Italy; katia.formica@irccsme.it (C.F.); francesco.corallo@irccsme.it (F.C.); viviana.lobuono@irccsme.it (V.L.B.)

**Keywords:** augmentative and alternative communication, brain injury, cognitive rehabilitation, quality of life

## Abstract

Background: Augmentative and Alternative Communication (AAC) improved communicative skills in adults with post-stroke aphasia demonstrating the effectiveness in speech disorders and consequent improvement of patients’ communication skills. This study aimed to report the efficacy of AAC in the rehabilitation of cognitive disorders and to estimate how the changes in cognitive and communicative functions could enhance the quality of life in patients affected by severe acquired brain injury. Methods: Three patients with pontine cerebral ischemia, traumatic brain injury (TBI), and meningioma expressed in the posterior cranial fossa, respectively, were submitted to rehabilitative training with AAC for 6 months. Patients underwent to neuropsychological and mood evaluations at the beginning of AAC treatment (T0) and after rehabilitative training (T1). Results: The results support the efficacy of AAC in the improvement of cognitive functions, particularly in memory, attention, and language domains. In addition, we described also an improvement in the quality of life and a decrease in depressive symptoms. Conclusions: The AAC seems to be an important rehabilitative technique for the recovery of cognitive functions with a consequent effect in improvement of psychological aspects and quality of life in patients with Acquired Brain Injury (ABI).

## 1. Introduction

Augmentative and Alternative Communication (AAC) was defined by the Speech Pathology Australia [1] as “a clinical and educational practice that provides communication strategies, techniques, and interventions with a range of communication limitations”. The “augmentative communication” refers to use of strategies which improve residual language abilities; instead, the “alternative communication” indicates the use of alternative techniques and strategies for speech language [2,3]. The AAC can be implemented with low and high technology systems; low tech indicating the use of pencil–paper instruments, alphabet boards, and Picture Communication Symbols (PCS) to communicate, while high tech means use of electronic and interactive boards equipped with software for communication [4,5]. The American Speech-Language-Hearing Association (ASHA) include AAC strategies such as facial expressions, gestures and signs, light technology, or electronic specialized speech generating devices such as a Lightwriter™ or the Grid Pad™ [6]. Currently, there are various devices and programs used for the rehabilitation of patients with speech disorders: communicators, computers with optical readers, and touch screens [7]. 

For example, the Grid Pad uses a touch screen and a ‘grid’ system that breaks words down into categories, which can be ordered by the user to form sentences that are relayed with a synthesized voice; the most modern models also have internet access and Bluetooth capabilities, so they can send and receive emails, text messages, and hook up to the user’s environment, allowing for remote control of lights. Electronic AAC options can be accessed through direct touch, switching and scanning, or other alternate access options, including eye gaze, head stick, or mouse control. In addition to specialized equipment designed specifically for this purpose, mainstream products, such as smartphones and tablets, can also run communication applications or link to alternate access options, such as a head-mounted laser pointer or mouse [8]. 

Communication is a dynamic process that creates and conveys a mutual understanding between two or more people. Communication is an essential element in human interaction. Adults who developed language deficits as a result of neurodegenerative disorders, stroke, or traumatic brain injury may benefit from communication support in addition to the more conventional restoration-based approaches to rehabilitation [9]. Several studies show communication difficulties in these patients are associated with negative psychological consequences, such as stress, fear, anger, and feelings of frustration, in addition to the presence of anxiety and depression [10]. 

Acquired Brain Injury (ABI) can lead to communication alterations which may impact different aspects of the communication system, including motor speech, written language, cognitive communication, and the expressiveness of the language. In addition, communication disorders affect all those aspects concerning social integration in terms of difficulties in topic generation and maintenance, taking turns in conversation, knowing how much and what to say, reading facial expressions, and understanding the underlying intent of the communication [6,7]. ABI patients with communication deficits require a range of interventions that involve compensatory communication strategies and also the development or application of augmentative and/or alternative communication systems [11,12]. 

Following an ABI, finding an efficient communication channel is important to avoid patient frustration and allow the operator to structure a rehabilitation process suited to the patient’s needs. Therefore, AAC methods and tools must be adapted to the characteristics of the people to whom they are applied and the team of professionals who use them [13]. The needs of the same patient may vary during hospitalization in rehabilitation, so the same tool can be indicated initially and not later, considering, for instance, contexts clinical data regarding clinical evolution and treatments.

McNaughton and Light declared the benefits of mobile technologies in communication abilities in patients with complex communicative difficulties [14]. It was shown that the use of high technology (AAC tools) improved communicative skills in adults with post-stroke aphasia, demonstrating the effectiveness of AAC in speech disorders and the consequent improvement of patients’ communication skills. Current literature shows that these kinds of treatments can be applied also in another neurological diseases, such as locked-in syndrome (LIS) [15], amyotrophic lateral sclerosis [16], and traumatic brain injury [17] to improve cognitive communication skills. Corallo et al. [18] showed the positive impact of AAC on the social ability, anxiety, depression, and quality of life in patients with LIS. Furthermore, other studies highlighted that the AAC system promotes speech production [19,20], permitting requests and expressing their own needs, increasing vocabulary, length, and complexity of expressed message. 

In this study, we described the role of AAC in improving cognitive functions and estimated if the changes in cognitive and communicative functions could enhance the quality of life (QoL) in three ABI patients in the sub-acute phase.

## 2. Methods

This study included three patients who were admitted to a rehabilitative program within 3 months after ABI (cerebrovascular disease, traumatic brain injury, and post-surgical meningioma exeresis) presented with speech, motor disorders, and cognitive impairment. The study was conducted on May 2023.

Given the importance of the evaluation phase in patient care, the need to carry out a preliminary assessment that primarily evaluates whether there are deficits that prevent the use of AAC in terms of cognitive rehabilitation is evident. For these reasons, we considered it necessary to verify the level of consciousness through LCF, verify the level of understanding with the administration of the Token Test, and, given the presence of the tracheal cannula that prevented the verbalization, we used an assessment tool that allowed us to evaluate the reasoning skills through a non-verbal test such as Raven’s Progressive Matrices (Table 1). All patients were evaluated at the time of admission to neurorehabilitation (T0) training to develop the adequate rehabilitative program and to maximize recovery. Indeed, the patients had to have the ability to comprehend, cooperate, and participate. A follow-up evaluation was performed after 6 months of training with AAC (T1). Neuropsychological assessment and AAC training were carried out by one neuropsychologist and one speech–language therapist. 

### 2.1. Assessment

Patients were evaluated through a battery of cognitive tests and clinical scales to assess global cognitive level and specific cognitive domains with the aim of using AAC for rehabilitation in order to improve motivation in rehabilitative training and QoL. The Level of Cognitive Functioning Scale (LCF) [21], Raven’s Progressive Matrices that evaluates abstract reasoning [22], and the Token Test for verbal comprehension [23] were used to verify whether the patients could be rehabilitated by AAC tool (Table 2). In addition, we used the Wisconsin Card Sorting Test [24] and Corsi Block-Tapping Task [25] to evaluate executive functions, and the Paced Auditory Serial Addiction Test [26] to evaluate auditory information progressing. These tests were used to assess cognitive domains that we treated with AAC. Baseline assessment was performed 1 month (±15 days) after brain injury. The clinical assessment consisted of the administration of the Short Form-36 Questionnaire for the assessment of Quality of Life (SF-36) [27] and the Beck Depression Inventory (BDI) [28] for depressive symptoms (Table 3). 

### 2.2. Patients

At baseline, all patients showed normal cognitive functioning framework and comprehension suitable for neurorehabilitation by AAC, as seen in Table 2. All the three patients given their written consent to participate to the study.

#### Below We Report a More Accurate Description for Each Case

Case 1: A 71-year-old man was hospitalized with a diagnosis of “pontine cerebral ischemia, encephalopathy, and arterial hypertension”; the Magnetic Resonance Imaging (MRI) examination showed left nucleus-capsular ischemia, chronic ischemia of the periventricular white matter, and pontine ischemia. At the neurological examination, the patient showed severe tetraparesis with mixed hypertone, parkinsonism syndrome, and dysphagia.

At baseline, the neuropsychological evaluation showed language alterations: spontaneous speech was markedly diminished and there was a loss of normal grammatical structure. The ability to repeat phrases was also impaired and the patient showed a deficit in reading and denomination. Writing capabilities were also damaged but verbal comprehension was preserved. The patients showed a deficit in capacity and rate of information processing and sustained and divided attention. He presented with depressive symptoms and he had a poor quality of life. 

Case 2: A 41-year-old man was hospitalized after a traumatic brain injury (TBI). The Computed Tomography (CT) examination showed “small hemorrhagic areas” and diffuse axonal damage (DAI) located in the sub-axial and deep bilateral parietal-frontal area, temporal medial right, and left portion of the midbrain, with outbreaks in the left cerebral peduncle and on the right parietal cortex. At the neurological examination, the patient showed severe right tetraparesis and dysphagia.

The patient’s cognitive profile at baseline (T0) highlighted alterations in visuospatial memory, in the capacity and rate of information processing, and sustained and divided attention. The patient showed also deficits in set-shifting and executive functions, such as planning and abstraction. The language was impaired in verbal production with denomination deficit and less verbal initiative, and the patient showed good comprehension.

Case 3: A 45-year-old man was hospitalized with a diagnosis of meningioma expressed in the posterior cranial fossa; a CT examination showed a left cerebellar hemispheric malacic area due to craniectomy. The patient showed pure motor syndrome, left tetraparesis, and dysphagia. An electroencephalographic examination showed also an epileptic crisis. At the first neuropsychological evaluation (T0), he showed multiple cognitive deficits in attention and visuospatial memory. The spontaneous speech was markedly deficient and he displayed less eye contact during communication, but he had preservation of general comprehension. His mood was depressed and there was a poor general condition of quality of life. The patient was often sleepy and, when he was awake, he presented poor collaboration. The patient followed a rehabilitative treatment with the communicator.

### 2.3. Rehabilitation Treatment

The AAC tool was constituted by a symbolic communicator with 100 characters, working on a common personal computer. The system was compounded by the software EUROVOCS SUITE (Jabbla, Lakeland, FL, USA) for communication, an adapter, and a fastening system. Communication way was based on the patient’s selection and choice of images shown on the computer screen. Images, with primary needs, were presented at a distance measured in centimeters to make them easier to use. Letters to compose words and sentences were presented in capitals for easy reading. Through the aid of two sensors placed on the two sides of the head, the patients were able to scroll and select the images through different head movements. 

All the three patients underwent cognitive rehabilitative treatment with the digital communicator. Sessions were planned ad hoc for each patient based on their clinical, cognitive, and psychological conditions. Initially, they performed training for attention and memory: 15 min a day for 5 days a week for 2 weeks. In a second step, training was 30 min a day for 5 days a week for 3 months. During rehabilitative training, special boards were used for communication, permitting the patient the select the topic of conversation.

The second case, however, had an endotracheal cannula, and the digital communicator was applied for alternative communication by using eye tracking through external sensors. Additionally, the other two patients accessed the communication system with eye tracking. The software allowed the different grids and activities present to be used through the eyes to communicate with the operator. 

Following a multidisciplinary approach, all three patients underwent motor training twice a day for 60 min for 3 months with a series of passive exercises to reduce spasticity propaedeutic training for improving gait and mobility. The sessions were aimed at sensorimotor skills relevant to basic daily functions, such as getting up from lying, sitting, rolling, and turning. Additionally, speech therapy was performed to improve dysphagia, vocal tone, breathing, and speech coordination. In addition, speech therapy included the care tracheostomy to reduce the risk of complications. The training was conducted twice a day for 60 min every session for 3 months. 

### 2.4. Statistical Analysis

To determine whether the observed improvements are beyond measurement error, thus whether possible improvements reflected true biological changes or psychometric noise, we also computed the Reliable Change Index (RCI) calculation for each outcome to recovery. This statistic, indeed, allows for detecting significant changes in neuropsychological test performance and a within-person comparison of test scores over time [29]. If the RCI score is 1.96 or less then the change is not considered to be reliable, and it possibly occurred just due to the unreliability of the measure.

## 3. Results

We described results for the efficacy of AAC in cognitive and mood improvement. After the rehabilitative treatment with AAC, scores of all patients showed an improvement in cognitive functions and a decrease in depressive symptoms, as viewable in Table 3. The RCI showed statistically significant changes in many test scores, as reported in Table 4. Notably, patients who underwent craniectomy showed an improvement in attentional and amnesic abilities, and all showed improvement in QoL and mood scores.

## 4. Discussion

AAC is an area of clinical practice that compensates for the impairment and disability patterns of individuals with severe communication disorders. Specifically, AAC can augment speech or provide an alternative method for communication by replacing speech and serves as a means to improve communicative success for those with speech intelligibility and/or language deficit. Therefore, AAC can be used in the rehabilitation of different neurological conditions. However, limited literature information exists about how best to support AAC by people with cognitive communication impairments secondary to ABI.

In this study, we describe the efficacy of AAC in cognitive and mood improvement in three patients presenting with different clinical and etiologic pictures. Patients executed exercises of recognition of alphabet letters, numbers, and mathematical symbols, nouns and phrases comprehensions, writing of simple sentences, and calculations. 

AAC is heavily used in treatment of speech–language deficits and allowed processing time reduction of written language, an improvement in selecting letters and in composing words/phrases, as well as an increase in the speed of numerical calculations [30]. In particular, in case 1, the patient was able to recognize and type only the alphabet letters and numbers, and then he was able to write more complex words and concise but effective sentences, as well as to perform simple arithmetic operations. After AAC therapy sessions, his communication skills were improved, such as recognition of alphabet letters, numbers, and mathematical symbols, exercises of nouns and phrases comprehension, and improved writing of simple sentences and calculations. Overall, the most important outcome we observed was the facilitation of improved AAC training in therapeutic compliance. Indeed, it seems that the use of the digital communicator allowed the patient to interact more easily with others, improving mood and collaboration. We observed that the AAC system allowed the third patient to express his own needs, adding more and more communicative elements in communication, such as using even simple pointing sounds and gestures.

In line with the literature, the patients after AAC training showed an enhancement in their cognitive performance, particularly in attention, mental processing, and memory skills [31,32,33]. The novelty was the use of AAC also in improvement of cognitive skills in patients with ABI. This tool seems to be paving the way for maximized cognitive training. As mentioned above, we obtained different results for all three cases. These findings depend on the etiologies of the ABI, which were different for all three patients. Consequently, the recovery during the rehabilitative program was not the same; moreover, the brain areas involved were different, which influenced the variety and severity of symptoms reported. Notably, the first patient showed a significant recovery in executive functions, whereas the second and third patients showed significant recovery in attention, memory, and visuospatial abilities. These results are probably related to the patients’ improved ability to communicate with the environment, although we cannot exclude the effect of spontaneous brain recovery that occurs in the first weeks after an acute event.

AAC was implicated not only in the recovery of cognitive functioning and the improvement of communication skills, but also played an important role in reducing depressive symptoms, improving tolerance towards dissatisfaction due to communication difficulties, and improving patients’ quality of life [14,34]. We also observed an improvement in mood disorder and QoL in all three cases. In fact, all three patients showed an increase in adherence and treatment compliance, as well as a reduction in depressive symptoms, which was observed in BDI and SF-36 scores (Table 3 and Table 4). Psychosocial and emotional aspects can also impact cognitive communication competence. In terms of language and communication disorders, they improved the verbal initiative, a more context-appropriate language, and facilitated better eye contact during communication, providing greater satisfaction from improved communicative effectiveness in asking for their needs. Mood alterations, such as depressive disorders may be associated with cognitive impairments in attention, working memory, information processing, executive functions, and processing speed [35]. 

Communication is a need for social interaction. When the subject lacks speech clarity, he reduces participation in daily social events. AAC training seems to be essential to increasing the patient’s quality of life, which became widely accepted in clinical assessment of speech, language, or communication deficits. However, in the improvement of cognitive functions, above all, quality of life is influenced by several factors, as well as motor and speech therapy, that positively impact general quality of life: in particular, the acquisition, even partially, of autonomy in performance activities of everyday life, such as eating, drinking, and talking better. Indeed, one of the main goals of modern medicine is to improve the quality of life of persons with a neurological illness, especially one that leads to language disturbances [36]. 

This study is limited by its small sample size of only three participants. For this reason, the results described have limited generalizability, and caution should be used in interpreting the results of inferential analysis. However, calculation of the RCI criterion allowed us to assess whether a change over time in an individual score (i.e., the difference score between two measurements over time) can be considered statistically significant, and thus to classify diagnostic changes among assessments with improvement or decline in cognitive abilities. Repeating the study with a larger sample of adults with language disorders is necessary to generalize the results obtained.

The limited literature existing about the use of AAC in neurorehabilitation for ABI patients represents a point of reflection for the realization of future studies. It would be necessary to extend this kind of research to realize guidelines for the rehabilitation of severe ABI patients. AAC compensates for communication deficits and ensures that patients retain the ability to communicate throughout their lives. 

## Figures and Tables

**Table 1:** Assesment of neuropsychological, quality of life, mood, and communication aspects.

TEST	Domains	Short Description	Focus	Cut-Off
Level of Cognitive Functioning Scale [21]	Cognitive functions	It systematically describes and categorizes a patient’s level of consciousness and cognitive and behavioral functioning through which the patient typically progresses.	To assess cognitive functioning in post-coma patients and the patterns or stages of recovery typically seen after a brain injury (for inclusion criteria).	8 levels describe: I—No response; II—Generalized; III—Localized; IV—Confused-agitated; V—Confused, inappropriate, non-agitated; VI—Confused-appropriate; VII—Automatic-appropriate; VIII—Purposeful-appropriate.
Raven’s Progressive Matrices [22]	non-verbal abstract reasoning skills	The test consists of increasingly difficult pattern-matching tasks. Items on all forms ask the examinee to identify the missing component in a series of figural patterns. The test can be individually or group administered.	Non-verbal tests to assess abstract reasoning and fluid intelligence (for inclusion criteria).	18
Token Test [23]	Auditory comprehension	The materials consist of tokens that differ in color, shape (squares and circles), and size (large and small). The examinee has to follow verbal instructions which increase in complexity from simple commands (e.g., “Touch a circle,” “Touch the red circle”).	To examine auditory comprehension deficits, by having patients respond gesturally to the tester’s verbal command (for inclusion criteria).	26.50
Wisconsin Card Sorting Test (WSCT) [24]	Executive functions	The test consists of two card packs having four stimulus cards and 64 response cards in each. Each card is presented with various geometric shapes in different colors and numbers. The participants are expected to accurately sort every response card with one of four stimulus cards through the feedback (right or wrong) given to them based on a rule.	To measure such higher-level cognitive processes as attention, perseverance, working memory, abstract thinking, and set shifting (for the assessment of cognitive abilities).	90.50
Corsi Block-Tapping Task [25]	Memory and Visuospatial abilities	It involves mimicking a neuropsychologist as they tap a sequence of up to nine identical spatially separated blocks. The sequence starts simple, usually using two blocks, but becomes more complex until the subject’s performance suffers.	To assess visuospatial short-term working memory (for the assessment of cognitive abilities).	3.5
Paced Auditory Serial Addiction Test (PASAT) [26]	Auditory Attention	The subject listens to a tape recording of digits presented one at a time. The task is to add each number to the one immediately preceding it. For example, the recording might present the numbers 1, 7, 5, and 4. The patient adds the first two numbers (1 + 7) and responds with the number 8. The patient then adds the second two numbers (7 + 5) and responds with the number 12.	To evaluate auditory information processing, calculation ability, and sustained and divided attention (for the assessment of cognitive abilities).	17.1
Short Form-36 Questionnaire (SF-36) [27]	Quality of life	It comprises 36 questions that cover eight domains of health: physical functioning (10 items), role limitations–physical (4 items), bodily pain (2 items), general health (5 items), vitality (4 items), social functioning (2 items), role limitations–emotional (3 items), and mental health (5 items).	Self-report to measure functional health and well-being (for the assessment of psychological aspects).	72
Beck Depression Inventory (BDI) [28]	Mood	It is composed of items relating to depressive symptoms such as hopelessness and irritability, cognitions such as guilt or feelings of being punished, as well as physical symptoms such as fatigue, weight loss, and lack of interest in sex.	Self-report to measure the severity of depressive symptoms (for the assessment of psychological aspects).	13

**Table 2:** Sample’s description at baseline.

Case	Age	Gender	Level of Cognitive Functioning Scale	Raven’s Progressive Matrices	Token Test
1	71	male	6	30	31.75
2	41	male	5	29	27.50
3	45	male	5	24	31.50

**Table 3:** Neuropsychological evaluation of the three patients at admission (T0) and after AAC treatment (T1).

	Wisconsin Card Sorting Test [24]		Corsi Block-Tapping Task [25]		PASAT [26]		Short Form-36 Questionnaire for the Assessment of QoL (SF-36) [27]		Beck Depression Inventory (BDI) [28]	
Case	T0	T1	T0	T1	T0	T1	T1	T1	T0	TI
1	37.20	57.30	4.50	6.20	4.70	7.25	40	75	33	16
2	81.50	79.40	3.75	6.25	3.80	6.75	50	80	42	14
3	34.20	37.10	3.50	5.80	4.00	7.00	30	78	39	13

**Table 4:** Reliable Change Index (RCI) for each domain to recovery by AAC.

Case	Wisconsin Card Sorting Test	Corsi Block-Tapping Task	PASAT	SF-36	Beck Depression Inventory
1	12.46 *	1.67	1.80	29.9 *	8.82 *
2	1.30	2.45 *	2.08 *	25.63 *	14.52 *
3	1.80	2.25 *	2.12 *	41.01 *	13.48 *

* Statistically significant T1-T0 change.

## Data Availability

Data sharing is not applicable to this article as no new data were created or analyzed in this study.
